# Gorlin Syndrome-Associated Basal Cell Carcinomas Treated with Vismodegib or Sonidegib: A Retrospective Study

**DOI:** 10.3390/cancers16122166

**Published:** 2024-06-07

**Authors:** Giulia Murgia, Luca Valtellini, Nerina Denaro, Gianluca Nazzaro, Paolo Bortoluzzi, Valentina Benzecry, Emanuela Passoni, Angelo Valerio Marzano

**Affiliations:** 1 Dermatology Unit, Fondazione IRCCS Ca’ Granda Ospedale Maggiore Policlinico, 20122 Milan, Italypaolo.bortoluzzi@policlinico.mi.it (P.B.); valentina.benzecry@policlinico.mi.it (V.B.); emanuela.passoni@policlinico.mi.it (E.P.); angelo.marzano@policlinico.mi.it (A.V.M.); 2 Oncology Unit, Fondazione IRCCS Ca’ Granda Ospedale Maggiore Policlinico, 20122 Milan, Italy; 3 Department of Pathophysiology and Transplantation, Università Degli Studi di Milano, 20133 Milan, Italy

**Keywords:** nevoid basal cell carcinoma syndrome, sonidegib, vismodegib, hedgehog inhibitors, Gorlin syndrome, PTCH mutation

## Abstract

**Simple Summary:**

Gorlin syndrome (GS) is a genetic disorder characterized by multiple basal cell carcinomas (BCCs) due to mutations in the hedgehog signaling pathway. Patients with GS may need dozens or even hundreds of surgical procedures in their lifetime, which can leave them severely scarred, deformed, and disfigured. In 16 patients with GS, we examined the effectiveness, safety, and length of response to oral hedgehog inhibitors. According to our retrospective study, sonidegib inhibited the growth of both newly diagnosed and pre-existing basal cell carcinomas more successfully and safely than vismodegib.

**Abstract:**

Nevoid basal cell carcinoma syndrome (NBCCS), also known as Gorlin syndrome (GS), is a genetic disorder characterized by the development of multiple cutaneous BCCs due to mutations in the hedgehog signaling pathway. The use of hedgehog pathway inhibitors—vismodegib and sonidegib—has emerged as a promising therapeutic strategy for managing BCCs in individuals with GS. In a retrospective study conducted between March 2012 and January 2024, a cohort of 16 Gorlin syndrome patients who received treatment with either sonidegib or vismodegib were analyzed. The primary objectives of the study were to evaluate the efficacy, safety profile, and duration of response to oral hedgehog inhibitors in this patient population. The study assessed various parameters, including the number of new BCCs that developed before and after treatment initiation, the duration and sustainability of treatment responses, as well as the incidence of adverse effects associated with hedgehog inhibitor therapy. The findings of the study revealed that sustained treatment with hedgehog inhibitors could effectively suppress the progression of both new and existing BCCs. Furthermore, the results indicated that sonidegib exhibited superior efficacy and safety compared to vismodegib in the treatment of BCCs in individuals with GS. Notably, adjustments to the administration schedule of sonidegib were found to improve tolerability without compromising therapeutic efficacy, potentially leading to prolonged durations of treatment response and disease control.

## 1. Introduction

Gorlin syndrome (GS), also known as Gorlin–Goltz syndrome, nevoid basal cell carcinoma syndrome, or basal cell nevus syndrome (BCNS), is an autosomal dominant familial cancer syndrome that is characterized by the early onset of multiple BCCs and/or mandibular odontogenic keratocysts (OKCs) [1]. Macrocephaly, frontal bossing, facial dysmorphism such as cleft lip/palate, and face milia are common characteristics in approximately 60% of patients. By the age of 20, over 90% of individuals develop ectopic calcification of the falx cerebri. Skeletal anomalies and palmar or plantar pits (asymmetrical, 2–3 mm in diameter, 1–3 mm in depth, and developing in the second decade) are described. Additional features include ocular anomalies, such as cataracts, colobomas, and microphthalmos, as well as lymphomesenteric cysts. A susceptibility to either benign or malignant tumors, such as fibrosarcoma, nephroblastoma, ovarian fibroma (often bilateral and calcified), meningioma, medulloblastoma, and cardiac papillary fibroelastoma, is reported [1].

Few investigations on GS prevalence have been conducted, as reviewed by Evans et al. [2]. The prevalence rate of 1:57,000 that is most frequently cited originates from research conducted in northwest England, UK, including four million people. There is a theoretical range of 1:30,827 to 1:164,000. GS appears to be more prevalent among Caucasians, despite the possibility of ascertainment bias associated with BCC development being less prevalent among other races. In line with expectations, autosomal dominant inheritance causes a similar incidence of Gorlin syndrome in both sexes.

About 20–30% of GS cases are caused by a de novo pathogenetic variation, whereas 70–80% of cases have a relevant family history.

Loss-of-function mutations in the tumor suppressor gene PTCH1 (9q22.1–q31), which codes for the sonic hedgehog ligand receptor, cause GS. The clinical presentation’s varying expressivity may be attributed to modifier genes (SUFU and PTCH2) and environmental exposure.

Several diagnostic criteria for GS have been proposed. It should be noted that no molecular testing is required to meet the diagnostic criteria. When a patient meets two major diagnostic criteria and one minor diagnostic criteria, or one major diagnostic criteria and three minor diagnostic criteria, a diagnosis of GS is established [2]. Kimonis et al. developed a similar series of diagnostic criteria [3]. To date, no study has been able to determine which set of diagnostic criteria offers the optimal balance between specificity and sensitivity.

The Consensus Statement from the First International Colloquium on Basal Cell Nevus Syndrome (BCNS) states that the following are diagnostic standards for GS [4].

Major criteria include BCC before the age of 20 or a significantly elevated number of BCCs compared to skin type and previous sun exposure; OKCs of the jaw before the age of 20; palmar or plantar pitting; lamellar calcification of the falx cerebri; medulloblastoma, usually desmoplastic; and first-degree family members with BCNS.

Minor criteria include rib abnormalities; macrocephaly; cleft lip and palate; ovarian or cardiac fibroma; lymphomesenteric cysts; ocular abnormalities (for example, strabismus, hypertelorism, congenital cataracts, glaucoma, or coloboma); and other skeletal malformations (for example, vertebral anomalies, kyphoscoliosis, short fourth metacarpals, or postaxial polydactyly).

A heterozygous germline PTCH1 or SUFU pathogenic or potentially pathogenic mutation may be found by molecular genetic analysis. Genetic confirmation validates a diagnosis if clinical signs are not totally evident. Mutations in PTCH2 have occasionally been reported in patients with NBCCS [5].

Patients with GS may need dozens or even hundreds of surgical procedures in their lifetime, which can leave them severely scarred, deformed, and disfigured [6].

Recent studies have identified pathogenic mutations in the hedgehog pathway, especially in “patched” (PTCH1 and PTCH2) genes, in Gorlin syndrome [7]. Vismodegib and sonidegib, known as “hedgehog inhibitors” (HHIs), are inhibitors of downstream signaling that target “smoothened” (SMO). These oral medications have demonstrated notable clinical efficacy in treating locally advanced or metastatic sporadic basal cell carcinomas [8,9]. This is because somatic (non-inherited) PTCH mutations are typically expressed in sporadic BCCs [10].

HHI drugs are associated with class-specific adverse effects (AEs), including dysgeusia (44–58%), fatigue (32–39%), hair loss (49–66%), muscular spasms (54–71% for sonidegib–vismodegib), and weight loss (44–56%). A post hoc analysis of the duration and severity of treatment-emergent adverse events (TEAEs) in patients receiving the two HHIs revealed that patients receiving sonidegib (200 mg) had a lower incidence of dysgeusia, alopecia, and muscle spasms than those receiving vismodegib (150 mg) [11]. Regarding treatment, there is a dearth of clinical evidence to support immunotherapy (particularly cemiplimab) in patients with GS, and there are few publications regarding the long-term efficacy and safety of HHIs in patients with this condition. Nevertheless, the most recent European consensus-based interdisciplinary guideline for the diagnosis and management of basal cell carcinoma recognizes multiple BCCs in patients with this syndrome as locally advanced BCCs, and treatment with Hedgehog inhibitors is advised for Gorlin patients who are not responsive to surgery or radiation therapy [12]. Based on these assumptions, we conducted a case study on 16 Gorlin patients (with clinical and genetic diagnoses) to detect differences between the two drugs in terms of efficacy, safety, and handling. It is imperative to enhance treatment compliance and minimize drug exposure in patients diagnosed with Gorlin syndrome, as they necessitate long-term therapy to prevent the onset of new BCCs.

## 2. Materials and Methods

### 2.1. Study Population

Our retrospective investigation included individuals with GS who received oral HHIs (vismodegib or sonidegib) between March 2012 and January 2024, identified by a search through our NMSC patient databases. The BCNS Consensus Statement defined diagnostic criteria for Gorlin syndrome. Patients taking vismodegib or sonidegib for other reasons, such as spontaneous basal cell carcinomas, were excluded from the study.

### 2.2. Study Methods

Records were collected in a spreadsheet format (Microsoft Excel version 16.77.1; Microsoft, Redmond, WA, USA) and comprised demographics, comorbid illnesses, and the type and the length of HHI treatment. The early response to HHIs was described, along with the TEAEs and drug response/resistance patterns. The number of BCCs before and during HHI therapy was determined using pathological examinations and dermatologist records. The time to progression was estimated from the beginning of HHI treatment up to the date of the histological documented recurrence. Each patient received a randomly generated patient number, and all patient information was de-identified prior to analysis.

### 2.3. Treatment Regimens

For many decades, individuals with GS were managed surgically, with several basal cell carcinoma excisions. Nowadays, if the number of BCCs is too large for appropriate surgical management, or if the patient is considered challenging to treat or unsuitable for radiation or surgical treatment after an evaluation by a multidisciplinary team, oral HHIs are prescribed.

### 2.4. Response Assessment

The effectiveness of HHI treatment was measured through a comparison of the number of BCCs after 4 months of HHI treatment with the number of lesions before therapy. A complete response was referred to as 100% clearance of all cutaneous BCCs. A partial response was described as a reduction in the size and number of existing BCCs, without any new lesions occurring. Progression was defined as the recurrent growth of BCCs or the formation of new BCCs. Time to progression (TTP) was determined by the occurrence of biopsy-proven new lesions while taking an HHI. Relapses were classified as local or generalized based on the size and location(s) of new or recurring BCCs. Localized resistance has been defined as the development of 1–3 new superficial resectable BCCs while being treated with HHI. Generalized resistance was defined as the occurrence of >3 new BCCs. Progression-free survival was defined as the period of time between the start of HHI and the first BCC recurrence.

### 2.5. Statistical Analysis

Statistical analyses were carried out using IBM SPSS statistics, version 29.0.1.0 (SPSS Inc., Chicago, IL, USA). Continuous variables were reported as medians and interquartile ranges (IQRs), according to their distribution, and were compared using non-parametric tests. Discrete variables were described as numbers and percentages and compared through Fisher’s exact test. The Kaplan–Meyer method was used to describe the progression-free survival (PFS) of the two treatments, vismodegib and sonidegib. Follow-up of PFS was presented as the mean ± standard error and median value if present. A *p*-value < 0.05 was considered statistically significant.

## 3. Results

We collected data from a total of 16 patients with a median age of 55 (interquartile range (IQR): 49.5–70.75). The median age of diagnosis expressed in years was 38 (25–57), while the median age of onset of symptoms was 16 (14–28). Our sample was almost equally divided by sex (eight males and eight females). All patients (100%) had a genetic mutation in PTCH1/PTCH2 or SUFU genes. Nine out of sixteen patients (56%) had an affected family member, and four out of these nine patients (44%) had two affected family members. All patients developed BCCs with a median age at diagnosis of the first BCC of 27 (17–35); in fact, only 5/16 (31.3%) developed the first BCC before the age of 20. As many as 15/16 (93.8%) had numerous BCCs not justifiable by photoexposure or phenotype. Pits were present in 10/16 (62.5%), with a median age at diagnosis of 30 (14–47.5). Keratocysts were present in 14/16 (87.5%), with a median age at diagnosis corresponding to 17 (14.50–25.75). Falx cerebri calcification was present in 6/16 patients (37.55%). Only 3/16 (18.75%) patients developed a medulloblastoma. Table 1 shows the patients’ major and minor diagnostic criteria and comorbidities.

### 3.1. Comparison of Vismodegib and Sonidegib

**Figure 1:**
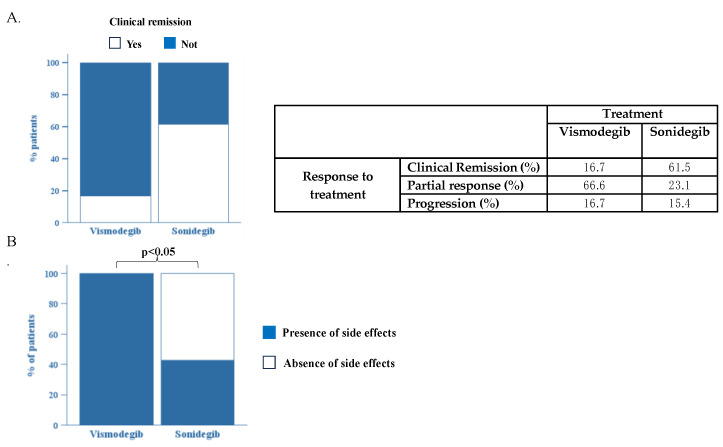
(**A**). Comparison of efficacy of treatment with vismodegib and sonidegib. (**B**). Comparison of patients who developed side effects during treatment between groups of patients treated with vismodegib and sonidegib. *p* < 0.05 via Fisher’s exact test.

In our samples, 10/16 patients received only sonidegib, 3/16 patients received vismodegib first and were later switched to sonidegib due to progression, and 3/16 patients received only vismodegib. After four months of HHI treatment, about 61.5% of the sonidegib patients achieved clinical remission, while only 16.7% of the vismodegib patients achieved it (Figure 1A). A partial response was obtained in 66.6% of patients with vismodegib and in 23.1% of those treated with sonidegib. After four months of treatment, 16.7% of patients on vismodegib and 15.4% of patients on sonidegib experienced a progression of the disease (Figure 1A).

Although the number of our samples was limited, the data we obtained show a clear superiority of sonidegib over vismodegib in terms of effectiveness, but large, confirmatory prospective clinical trials are needed.

Interestingly, our analysis highlights the superiority of sonidegib over vismodegib in term of safety. In fact, all (100%) patients treated with vismodegib developed at least one side effect compared to only 57.9% of the patients on sonidegib (*p* < 0.05) (Figure 1B).

**Figure 2:**
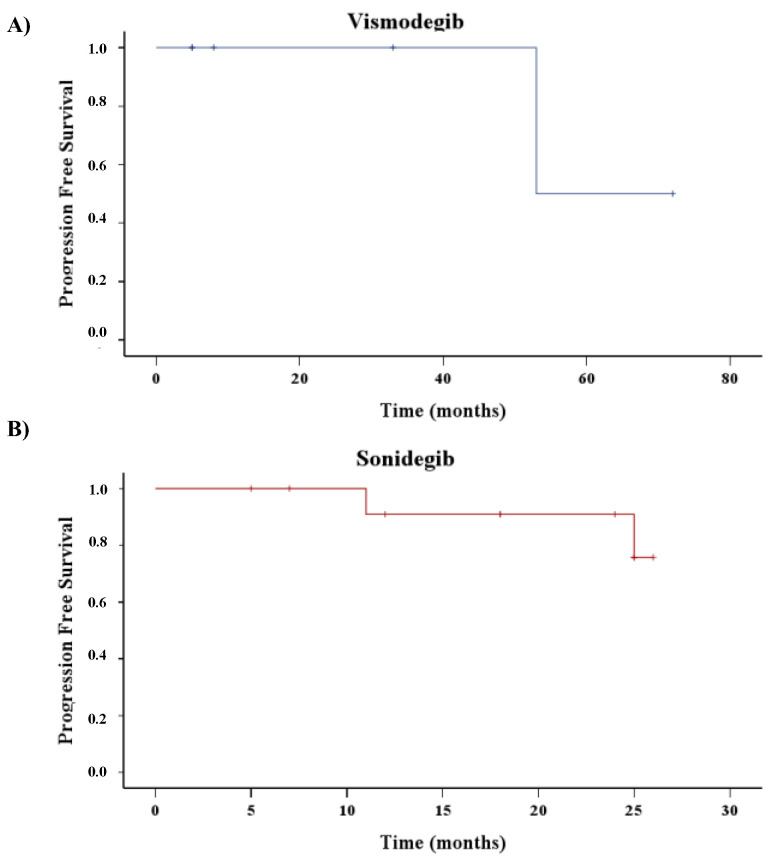
Time to first new BCC recurrence from the beginning of HHI treatment. Hash marks represent censored patients. Kaplan-Meyer curves of patients treated with Vismodegib (**A**) or Sonidegib (**B**).

The median of follow-up for patients treated with vismodegib (*n* = 6) was 20.5 months (5.0–57.75), and, of those, three out of six stopped treatment and three out of six switched to sonidegib. The median of follow-up for patients treated with sonidegib was 21 months (11.75–25), and all patients are still under treatment. It is interesting to note that in the sonidegib group, the median of PFS was not reached; thus, during all the time of surveillance of these patients, more than 50% were free from progression (Figure 2B). On the contrary, it should be recorded that in the vismodegib group (Figure 2A), the Kaplan–Meyer curve showed a median PFS of 53.0 months.

### 3.2. Association between Response to Sonidegib and Clinicopathological Features

We then evaluated whether or not any clinical features present at baseline could be associated with the therapeutic response to sonidegib (Supplementary Table S1). According to our analysis, patients who experienced clinical remission following 4 months of sonidegib treatment had BCC counts < 100 (*p* < 0.05), fewer than 4 BCC counts annually (*p* < 0.05), and fewer than 2 disease recurrences in high-risk locations (*p* < 0.05).

Photo S1 illustrates clinical and histological remission of laBCC after three months of Sonidegib.

## 4. Discussion

The maintenance of somatic stem and pluripotent cells as well as the development of different organ systems are crucial functions of the three human “hedgehog” (HH) peptide family members: Sonic, Desert, and Indian Hedgehog [13]. These soluble proteins have varying tissue-regulated patterns of expression, which account for their apparent functional variations. The binding of all three HH isoforms to the 12-pass transmembrane receptor “patched” (PTCH) allows them to function. Two PTCH homologs are currently known in mammals: Patched1 (PTCH1) and Patched2 (PTCH2). Each isoform has a tissue-specific expression, regulates different cellular development patterns by binding all three HH peptides with equal affinity, and suppresses SMO activity. Mutations within PTCH genes have been identified in Gorlin syndrome and sporadic basal cell carcinomas. Medulloblastomas are typically related to “suppressor of fused homologue” (SUFU) gene modifications [1].

BCC carcinogenesis is ligand-independent because the HH pathway is constitutively activated by changes in its components, involving gain-of-function (GOF) mutations in the SMO gene and loss-of-function (LOF) mutations in the PTCH1 or SUFU genes [1].

Approximately 90% of individuals with sporadic BCC have a detectable monoallelic LOF mutation in PTCH1, 30% have biallelic inactivation, and 10% have GOF mutations in SMO. The majority of sporadic BCCs have increased expressions of the proteins PTCH1 and GLI [14].

In patients with GS, a monoallelic PTCH1 mutation on chromosome 9 is frequently identified. It is noteworthy that 27% of individuals with clinically diagnosed Gorlin do not exhibit any detectable mutation in either PTCH1 or SUFU, suggesting the possibility of further undiscovered causative mutations [1].

Cyclopamine was the first hedgehog inhibitor to be developed. This drug suppresses downstream hedgehog signaling by binding to SMO [15]. Nevertheless, cyclopamine causes quite serious birth defects. Safer synthetic equivalents of cyclopamine have been developed and approved: sonidegib (LDE225) and vismodegib (GDC-0449).

These new oral HHIs have demonstrated effectiveness and safety in the management of laBCC. When HHIs bind selectively to SMO, the hedgehog pathway is deactivated. As reported by Dummer et al. [16], the ORRs of HHIs for laBCC were recorded as 47.6% for vismodegib over a 21-month follow-up period and 60.6% for sonidegib during an 18-month follow-up period. At the 30-month follow-up, the centrally evaluated mDOR for sonidegib was 26.1 months, and at the 21-month follow-up, it was 9.5 months for vismodegib. At the 30-month follow-up, the centrally evaluated mPFS for sonidegib was 22.1 months, whereas at the 21-month follow-up, it was 9.5 months for vismodegib. A review of published data from both pivotal trials (ERIVANCE and BOLT) showed that sonidegib exhibited around a 10% lower incidence of most adverse events (AEs) compared to vismodegib. In general, TEAEs associated with sonidegib were slightly less common and milder than those associated with vismodegib. Except for fatigue, the time to onset of AEs for individuals treated with sonidegib suggested that AEs may occur slightly later in relation to vismodegib.

There are just a few case reports and three published case studies including individuals with Gorlin syndrome who received HHI treatment in the literature [17,18,19,20,21,22,23,24,25,26,27]. All these studies showed a decrease in the quantity and size of BCCs, along with a rapid onset of response. The effectiveness and tolerance of long-term therapy have not been extensively investigated.

In another real-world experience with sonidegib, Nazzaro et al. documented 11 patients, 4 of whom were diagnosed with GS [27]. Of the patients, seven (63.6%) had adverse events (AEs); however, only three had their medication stopped because of toxicity. Four patients (50%) had complete remission (CR) confirmed by biopsy, while three patients (37.5%) had a partial response (PR). In total, 12.5% of the patients had a stable illness (SD). Sonidegib treatment was able to manage the disease in all four of the GS patients. Numerous novel HHI agents have been investigated as a result of vismodegib’s and sonidegib’s activity in BCCs [28,29]. These include topical medications like patigedib and medications that target mutations that cause drug resistance (taladegib, TAK441, and LEQ506). Cemiplimab, a PD-1-directed monoclonal antibody, has also demonstrated notable therapeutic effectiveness against sporadic BCCs [30]. The relevance of these newer medications in Gorlin disease therapy is still being investigated.

The main limitation of our retrospective study is the small sample size for a rare disorder. Furthermore, the number of BCCs identified before therapy varied significantly across individuals. A bias in lead time might have arisen from this. Additionally, there was a chance of ascertainment bias when determining the quantity of BCCs before and after therapy. It is possible that tumors were excised or eliminated without a histological examination, as is common with non-melanoma skin cancer. Further, data were sometimes unavailable for more than 5–7 years due to the extended duration during which individuals were susceptible to developing BCC. Furthermore, the patients in our study switched doctors often, making it challenging to collect all previous biopsy reports.

## 5. Conclusions

Our retrospective investigation assessed the efficacy and safety of HHIs in treating 16 individuals with clinically and genetically confirmed GS. After four months of HHI treatment, about 61.5% of sonidegib patients and just 16.7% of vismodegib patients achieved clinical remission. A partial response was obtained in 66.6% of patients treated with vismodegib and in 23.1% of those treated with sonidegib. According to our analysis, patients who experienced clinical remission following 4 months of sonidegib treatment had BCC counts < 100, fewer than 4 BCC counts annually, and fewer than 2 disease recurrences in high-risk locations. Interestingly, our analysis highlights the superiority of sonidegib over vismodegib in term of safety. In fact, all (100%) patients treated with vismodegib developed at least one side effect compared to only 57.9% of patients on sonidegib (*p* < 0.05). Studies of the pharmacokinetic profiles point out that sonidegib seems to be more lipophilic than vismodegib, with a volume of distribution of >9.000 L, indicating extensive distribution in tissues, while vismodegib has a volume of distribution of 16–27 L, suggesting that it is largely confined to the plasma. In theory, this evidence indicates that sonidegib is more distributed in the skin compared with vismodegib, which may potentially explain the differences in efficacy and toxicity observed in our case series [31].

Since the use of long-term HHI medication in patients with GS appears to be extremely effective, future research with longer follow-ups should investigate HHIs’ effectiveness and safety in these patients. Our case series proved the superiority in terms of effectiveness and safety of sonidegib over vismodegib in the treatment of BCCs in Gorlin syndrome. These data, together with the option of dose interruptions, supportive medications to better manage the adverse events, and on-label dose reduction for sonidegib to the “every other day” schedule [32,33], make this molecule a candidate for the long-term management of these chronic patients and one to be investigated with large, controlled trials. Since drug resistance may be caused by mutations that confer resistance to sonidegib or vismodegib [21,34], the development of new HHIs may also be significant. Resection of possibly resistant BCCs is advised in patients with a small number of progressing lesions, as it may extend the duration of the benefit of HHI treatment.

## Figures and Tables

**Table 1:** Patient characteristics.

UPN	Age	Sex	Major Criteria	Minor Criteria	BCC Histologic Subtypes	Genotype	Comorbid Conditions	HHI Treatment	Clinical Response	TEAEs
1	55	M	>100 BCCs; Palmar pits	Frontal bossing; Macrocephaly; Cleft lip/palate	Nodular superficial, infiltrating, pigmented, sclerosing	PTCH1 T230K	/	Vismodegib; Sonidegib	Vismodegib: progression Sonidegib: complete response	With vismodegib, alopecia and muscle cramps
2	55	M	>100 BCCs; OKCs; Falx cerebri calcification	Frontal bossing; Macrocephaly	Nodular, superficial, pigmented	PTCH1 Q196X	/	Sonidegib	Complete response	Fatigue
3	31	F	>100 BCCs; First-degree relative with BCNS	Frontal bossing; Macrocephaly; Bifid ribs; Scoliosis	Nodular, micronodular, superficial, infiltrating, basosquamous	PTCH1 L106R	/	Sonidegib	Partial response	Dysgeusia
4	81	F	100 BCCs; OKCs; Medulloblastoma	Macrocephaly; Cleft lip/palate; Scoliosis	Nodular, superficial, infiltrating, pigmented, adenoid, sclerosing	PTCH1 mutation c.3397A>5 (p.T1133A) exon 20	Hypercholesterolemia; IPMN	Vismodegib	Partial response	Muscle cramps; Alopecia; Dysegeusia
5	48	F	50 BCCs; OKCs; Falx cerebri calcification; First-degree relative with BCNS	Macrocephaly; Pectus deformity	Nodular, superficial, pigmented, sclerosing	PTCH1 mutation c.3397A>5 (p.T1133A) exon 20	/	Sonidegib	Complete response	/
6	49	M	>100 BCCs; OKCs; Palmar pits; First-degree relative with BCNS	Macrocephaly; Kyphoscoliosis; Cleft lip/palate	Nodular, superficial, infiltrating, pigmented, adenoid	PTCH1 c.2908 G>T (p.E970X) exon 18	/	Vismodegib	Partial response	Alopecia; Dysgeusia; Weight loss
7	51	F	50 BCCs; OKCs; Palmar pits Falx cerebri calcification; First-degree relative with BCNS	Frontal bossing; Macrocephaly; Ovarian fibromas	Nodular, superficial, pigmented, sclerosing	PTCH1 c.3027 C>G (p.Y1009X)	/	Vismodegib; Sonidegib	Vismodegib: partial response Sonidegib: partial response	With vismodegib, dysgeusia
8	77	M	40 BCCs; OKCs	Fused ribs; Hemivertebra; Kyphoscoliosis; Pectus deformity; Glaucoma	Nodular, superficial, pigmented	PTCH1	Hypertension; Dyslipidemia	Vismodegib; Sonidegib	Vismodegib: partial response Sonidegib: partial response	With vismodegib, alopecia
9	63	M	35 BCCs; OKCs; Palmar pits; First-degree relative with BCNS	Macrocephaly; Kyphoscoliosis; Cleft lip/palate	Nodular, superficial, pigmented	PTCH1	/	Sonidegib	Complete response	/
10	72	F	33 BCCs; OKCs; Palmar pits; Medulloblastoma	Frontal bossing; Ovarian fibromas; Hypertelorism	Nodular, superficial, pigmented, infiltrating	PTCH1	Hodgkin lymphoma	Sonidegib	Complete response	Fatigue
11	67	F	16 BCCs; OKCs; Palmar pits; First-degree relative with BCNS	Macrocephaly; Kyphoscoliosis; Cleft lip/palate; Strabismus	Nodular, superficial, pigmented	PTCH1	/	Sonidegib	Complete response	Dysgeusia
12	54	M	>40 BCCs; OKCs; Falx cerebri calcification; First-degree relative with BCNS	Frontal bossing; Ovarian fibromas; Scoliosis	Nodular, superficial, pigmented, sclerosing	PTCH1	/	Vismodegib	Complete response	Muscle cramps
13	42	F	45 BCCs; OKCs; Palmar pits; Falx cerebri calcification	Macrocephaly; Cleft lip/palate	Nodular, superficial, pigmented	PTCH1	/	Sonidegib	Complete response	Weight loss
14	79	F	40 BCCs; OKCs; Palmar pits; First-degree relative with BCNS	Frontal bossing; Kyphoscoliosis; Cardiac fibroma	Nodular, superficial, infiltrating	PTCH1	Oval foram pervium; Atrial fibrillation	Sonidegib	Complete response	/
15	54	M	>50 BCCs; OKCs; Falx cerebri calcification; Palmar pits; First-degree relative with BCNS	Macrocephaly	Nodular, superficial, basosquamous, infiltrating	PTCH1 c.2536_2537insG (p.His846ArgfsTer15)	Hypertension	Sonidegib	Partial response	Alopecia
16	64	M	>1000 BCCs; OKCs; Palmar pits; Medulloblastoma	Cataracts and glaucoma	Nodular superficial, infiltrating pigmented, sclerosing	SUFU deletion c.1016	Myocardial infarction; Multiple trichoepithelioma	Sonidegib	Progression	/

UPN: unique patient number; OKCs: odontogenic keratocysts; IPMN: intraductal papillary mucinous neoplasia of the pancreas.

## Data Availability

The de-identified data underlying this case series will be shared upon reasonable request to the corresponding authors.

## Supplementary Materials

The following supporting information can be downloaded at: https://www.mdpi.com/article/10.3390/cancers16122166/s1. Supplementary Table S1: Baseline clinicopathological features of patients treated with sonidegib predicting clinical remission. Photo S1: (A) Clinical and histological presentation of laBCC before sonidegib treatment. (B) Clinical and histological remission after three months of sonidegib.
