# Supplementary material for: Gorlin Syndrome-Associated Basal Cell Carcinomas Treated with Vismodegib or Sonidegib: A Retrospective Study
Source: Cancers (Basel). 2024 Jun 7;16(12):2166. doi: 10.3390/cancers16122166 (PMC11201977; doi:10.3390/cancers16122166)

Supplementary Table S1. Baseline clinicopathological features of patients treated with sonidegib predicting clinical remission.

	Clinical remission		
	**Yes (*n*=8)**	**No (*n*=5)**	***p value***
**Female (%)**	62.5	20.0	0.27*
**Age (median (IQR))**	58.5 (27)	55.0 (11)	0.83^{+}
**Age of symptoms onset:**			
**median (IQR)**	15.5 (7)	28.0 (16)	0.065^{+}
**≤ 20 years (%)**	87.5	40.0	0.22*
**Age of BCC onset:**			
**median (IQR)**	25.0 (17.7)	28.5 (8.2)	0.68^{+}
**≤20 years (%)**	37.5	0.0	0.23*
**Great number of BCC not justified by photo exposition or phenotype (%)**	100.0	80.0	0.38*
**Number of BCC ≥100 (%) ****	0.0	60.0	***0.03****
**Number of BCC/year ≥4 (%) ****	0.0	100.0	***0.01****
**FH BCC (%)**	87.5	60.0	0.51*
**Presence of comorbidity (%)**	50.0	20.0	0.56*
**Pits (%)**	62.5	80.0	1.0*
**Age of Pits onset (median (IQR))**	30.0 (24.5)	43.0 (42.5)	0.56^{+}
**Presence of keratocysts (%)**	100.0	60.0	0.13*
**Presence of bilamellar calcification (%)**	25.0	40.0	1.0*
**Presence of rib abnormalities (%)**	12.5	0.0	1.0*
**Presence of macrocephaly (%)**	62.5	60.0	1.0*
**Presence of labiopalatoschisis (%)**	0.0	40.0	0.15*
**Presence of ovarian fibroids (%)**	25.0	20.0	1.0*
**Presence of eye problems (%)**	25.0	0.0	0.48*
**Presence of recurrent BCC (%)**	62.5	100.0	0.23*
**Number of recurrences (median (IQR))**	1.0 (2.75)	3.0 (3)	0.09^{+}
**Number of recurrences in high-risk areas (median (IQR))**	0.5 (1.0)	3.0 (3.5)	***0.04***$^{+}$

**Number of BCC with local invasion (median (IQR)**	1.0 (1.5)	1.5 (1.75)	0.68^{+}

*p-value derived from Fisher's exact test; +p-value obtained by Mann Whitney test.

** BCC diagnosed prior to sonidegib introduction

Abbreviation: IQR, Interquartile range; BCC, basal cell carcinoma; FH, family history.

Photo S1. Clinical and hystological presentation of laBCC before Sonidegib treatment. **1B** Clinical and histological remission after three months of Sonidegib.

A.

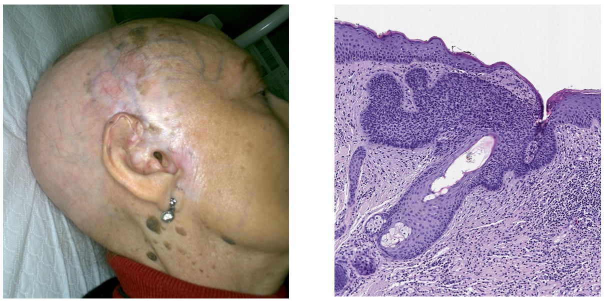

B.

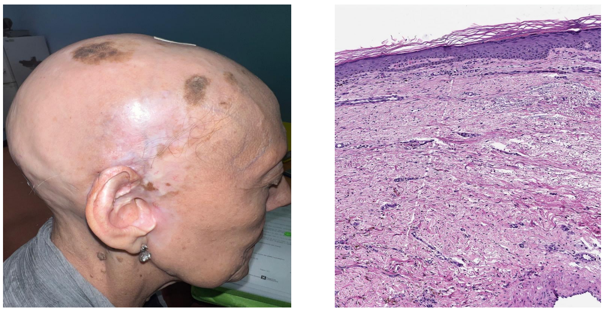

Supplement: Supplementary file 1 [file cancers-16-02166-s001.zip › cancers-3022178-supplementary.pdf]
